# Seasonal and Age-Stratified Patterns of Influenza, COVID-19, and RSV in Poland (2024–2026)

**DOI:** 10.3390/v18091038

**Published:** 2026-09-19

**Authors:** Wiktoria Majewska, Barbara Poniedziałek, Lidia B. Brydak, Andrzej Fal, Piotr Rzymski

**Affiliations:** 1 Department of Environmental Medicine, Poznan University of Medical Sciences, 60-806 Poznan, Poland; wiktoria.majewska@ump.edu.pl (W.M.); bpon@ump.edu.pl (B.P.); 2 Doctoral School, Poznan University of Medical Sciences, Fredry St. 10, 61-701 Poznan, Poland; 3 Department of Virology, National Institute of Public Health, NIH-National Research Institute, 00-791 Warsaw, Poland; lbrydak@pzh.gov.pl; 4 Faculty of Medicine, Collegium Medicum, Cardinal Stefan Wyszynski University, 01-938 Warsaw, Poland; amfal@wp.pl

**Keywords:** respiratory infections, respiratory syncytial virus, RSV, influenza, SARS-CoV-2, COVID-19, epidemiology, public health

## Abstract

Respiratory syncytial virus (RSV), influenza viruses, and severe acute respiratory syndrome coronavirus 2 (SARS-CoV-2) remain major causes of seasonal respiratory morbidity, yet their contemporary temporal relationships and age-specific dynamics are insufficiently characterized. We analyzed nationwide Polish surveillance data for the 2024/25 and 2025/26 epidemic seasons, reporting the weekly infection burden per 100,000 population, both overall and by age group (0–4, 5–19, 20–59, and ≥60 years). The seasonal magnitude, peak timing, peak width, normalized temporal trajectories, and age-specific lead–lag patterns were compared. In both seasons, respiratory virus circulation followed the same temporal sequence: COVID-19 activity increased first and peaked in September, followed by influenza in January/February and RSV infections in February/March. Mean weekly burden was lower in 2025/26 for all three infections, by 22.0% for influenza, 19.5% for COVID-19, and 16.0% for RSV, with the between-season difference reaching statistical significance only for influenza (*p* = 0.0024). Age-stratified analyses likewise showed significantly lower influenza burden in 2025/26 across all age groups, whereas for COVID-19, a significant reduction was observed only among adults aged ≥60 years; no significant between-season differences were found for RSV in any age group. Despite the lower mean influenza burden, the 2025/26 season produced a higher, narrower influenza peak, while the COVID-19 and RSV peaks were lower. Temporal trajectories were highly concordant between seasons for influenza (Spearman’s Rs = 0.975) and RSV infections (Rs = 0.956) and to a lesser extent for COVID-19 (Rs = 0.883). Age-specific temporal trajectories were strongly concordant across age groups, particularly for influenza (pairwise same-week correlations (Rs = 0.972–0.987 in 2024/25 and Rs = 0.963–0.992 in 2025/26). Influenza activity was largely synchronous across age groups, with pediatric peaks occurring approximately 1 week earlier, whereas COVID-19 and, to a lesser extent, RSV showed more consistent pediatric leads relative to adults aged ≥60 years (approximately 1–2 weeks). However, for all three infections, the ascending phase became apparent earlier in children aged 0–4 years than in adults, suggesting that peak timing alone may underestimate the pediatric lead. These findings support integrated, age-stratified surveillance and indicate that preventive strategies, particularly updated COVID-19 vaccination, should be timed to precede the early-autumn rise in transmission.

## 1. Introduction

Respiratory viral infections continue to pose a major public health challenge worldwide, especially in developed countries [1,2]. Among them, respiratory syncytial virus (RSV), influenza viruses type A and B, and severe acute respiratory syndrome coronavirus 2 (SARS-CoV-2) are the key drivers of seasonal morbidity and healthcare utilization [3,4,5,6]. Importantly, although these viruses increasingly co-circulate and overlap within the same annual surveillance periods, they differ substantially in their seasonality, age distribution, predictability of epidemic activity, and opportunities for prevention. Understanding these differences, as well as the temporal relationships between their epidemic waves, has become particularly important in the post-COVID-19 pandemic period [7].

Influenza is characterized by well-established seasonality, but its epidemic behavior remains difficult to predict due to antigenic drift and shift [8], which contribute to variable vaccine effectiveness and to epidemics of differing intensity and severity, especially among older adults and individuals with comorbidities [8,9,10,11]. In addition, the COVID-19 pandemic period and subsequent changes in population immunity and healthcare-seeking behavior have altered influenza’s epidemic dynamics, with irregular, delayed, or more intense peaks observed in several regions, including Europe [11,12,13]. These shifts underscore the ongoing need to closely monitor influenza circulation patterns and reassess vaccination timing and coverage strategies in light of post-pandemic behavioral and virological changes.

RSV represents another strongly seasonal respiratory infection, although its epidemiological profile differs substantially from that of influenza. While it has long been regarded primarily as a pediatric pathogen, growing evidence highlights its significant disease burden in adults and older individuals [14,15]. In these populations, RSV can cause severe lower respiratory tract infections, often indistinguishable from influenza or COVID-19. However, the awareness of RSV as a serious threat beyond childhood remains low, or at least lower than for other respiratory infections, both among patients and healthcare professionals, potentially contributing to underdiagnosis and underestimation of its true impact [16,17,18]. At the same time, broader recognition of RSV burden and increasing access to diagnostic testing have changed the surveillance landscape. Although molecular methods remain the diagnostic gold standard, wider use of rapid diagnostic assays in outpatient and emergency settings may facilitate recognition of RSV infections across a broader range of patients [19,20,21]. Consequently, contemporary surveillance data may provide a more complete picture of RSV activity across age groups than was possible in the pre-pandemic period.

Compared with the relatively established seasonal patterns of influenza and RSV, the temporal behavior of SARS-CoV-2 remains considerably less stable. Its timing and intensity continue to evolve under the combined influence of waning population immunity, viral evolution, and vaccination uptake [22]. Notably, the emergence and global dominance of the Omicron lineage and its subsequent descendants marked a pivotal shift in the epidemiology of COVID-19, with generally reduced clinical severity but substantially increased transmissibility and immune escape [23,24,25]. Although this shift has reduced the average individual risk of severe outcomes compared with earlier pandemic phases, the high transmissibility of Omicron subvariants continues to burden public health systems through large numbers of infections, population-level morbidity, workforce disruption, and the risk of long-term sequelae such as long COVID, particularly among vulnerable groups [26,27,28]. The timing of SARS-CoV-2 circulation also has direct implications for prevention. In Poland, updated COVID-19 vaccines are typically introduced in October–November; therefore, determining whether this timing corresponds to the actual epidemic pattern is essential for ensuring protection during periods of greatest transmission.

Taken together, these developments have created new opportunities to examine the epidemiology of major respiratory viruses in Poland more comprehensively. National surveillance of RSV was introduced relatively recently, beginning with the 2024/2025 epidemic season, while surveillance of influenza and COVID-19 has been incorporated into the same system, enabling simultaneous tracking of all three major respiratory viruses [29]. This integrated surveillance framework allows their epidemic trajectories to be compared within the same population and time periods, including differences in the timing and sequence of epidemic waves as well as age-specific patterns of circulation. Such analyses may provide a more comprehensive understanding of how these infections collectively shape the respiratory virus season and may help identify reproducible temporal relationships relevant to surveillance, preparedness, and the timing of preventive interventions.

Therefore, in this study, we aimed to compare the timing, magnitude, and age-specific temporal patterns of recorded influenza, COVID-19, and RSV cases in Poland during the 2024/25 and 2025/26 epidemic seasons. Specifically, we assessed (i) the sequence and timing of epidemic peaks, (ii) between-season similarity in normalized weekly trajectories, and (iii) whether age-specific patterns suggested reproducible temporal leads or lags relative to adults aged ≥60 years. We hypothesized that COVID-19 activity would precede influenza and RSV activity and that pediatric activity would precede activity in older adults for RSV and COVID-19. Results of our analysis may improve understanding of the burden and temporal dynamics of major respiratory infections across age groups and support preparedness for future epidemic seasons, including more appropriately timed vaccination strategies.

## 2. Materials and Methods

### 2.1. Data Source

The raw data for this study were obtained from the e-Health Center (Centrum e-Zdrowia) database on infectious diseases in Poland. It is a collaborative effort of the e-Health Center, the Chief Sanitary Inspectorate, and the Ministry of Health, ensuring the reliability and comprehensiveness of the dataset used in this study. This national database collects and integrates information on selected infectious diseases, including influenza, COVID-19, and respiratory syncytial virus (RSV). Data sources include medical events reported by healthcare providers to the national electronic medical database (the P1 system) and social insurance sick leave records, supplemented with demographic information. The database provides case counts standardized per 100,000 population.

### 2.2. Population and Study Period

The study population comprised the entire Polish population. Analyses focused on the 2024/25 and 2025/26 seasons, reflecting the post-pandemic period and the full integration of RSV diagnostics into the national system. The 2023/24 season was excluded due to incomplete reporting of RSV infections. Each epidemic surveillance season was defined as a standard annual cycle used by public health agencies to track seasonal respiratory illnesses, running from week 31 of one year to week 30 of the following year [30]. Data were stratified by patient age into the following four groups: 0–4 years, 5–19 years, 20–59 years, and ≥60 years. Because this was a population-level surveillance study, not a recruited cohort, there was no fixed study sample or participant flow. The analytical data consisted of weekly population-based case rates standardized per 100,000 inhabitants for the overall population and the predefined age groups. Individual-level demographic and clinical characteristics beyond the variables available in the surveillance extract were not part of the analytical dataset.

### 2.3. Case Definition and Inclusion Criteria

For RSV infection, cases were identified based on medical events coded as J12.1, J20.5, J21.0, or B97.4 according to the ICD-10 classification [31]. COVID-19 cases were defined by combining medical events and Polish Social Insurance Institution (ZUS) sick leave records with ICD-10 code U07 (including extensions), as well as confirmed positive tests from the nationwide EWP system, which monitors epidemic threats, quarantine, and isolation status in Poland. Influenza cases were defined by medical events and ZUS sick leave with ICD-10 codes J10 and J11 (including extensions).

Importantly, case ascertainment in the present study was based on these routinely collected diagnostic codes and data sources rather than on a requirement for a specific diagnostic test imposed by the study investigators. The source dataset did not provide information on the diagnostic method underlying each individual ICD-coded medical event or sick leave record; consequently, it was not possible to distinguish cases diagnosed using rapid antigen testing from those diagnosed using molecular testing or other diagnostic procedures. This distinction is particularly relevant for influenza, as ICD-10 code J10 denotes influenza due to an identified influenza virus, whereas J11 denotes influenza in which the virus was not identified. For COVID-19, positive tests recorded in EWP represented test-confirmed infections, whereas U07-coded medical events and sick leave records constituted an additional source of case identification. Similarly, the RSV-specific ICD-10 codes identify RSV as the etiological agent, but the database does not specify the diagnostic technique used to establish that diagnosis. Therefore, the cases analyzed here should be interpreted as recorded cases identified according to the national e-Health Center case-definition algorithm, rather than exclusively as infections confirmed by a particular laboratory or point-of-care testing method.

Each occurrence of a qualifying event was counted as a single case unless it occurred within 60 days of a previous qualifying event for the same infection in the same patient. The interval provides a conservative washout period intended to group multiple healthcare and administrative records potentially related to the same disease episode and thereby reduce overcounting resulting from repeated encounters or records. Similar 60-day windows have been used in analyses of routinely collected healthcare data to distinguish respiratory infection episodes [32,33]. The case date was assigned based on the first event not excluded by this rule.

### 2.4. Ethical Considerations

This study was based exclusively on the secondary analysis of fully anonymized epidemiological data routinely collected within the Polish national infectious disease surveillance system and submitted to the online eZdrowie database in Poland. The data had been collected independently of the present study for administrative and public health surveillance purposes and were not generated through any research-specific procedure. The investigators did not recruit or contact patients, perform any diagnostic or therapeutic intervention, modify patient care, or have access to information permitting the identification of individual patients. Accordingly, the study did not constitute a medical experiment involving human participants as defined by Polish law and did not require approval from a bioethics committee (Dz.U. 2020 poz. 1291). As the analysis was conducted solely using previously collected anonymized surveillance data, deposited in an online database, with no direct involvement of patients and no possibility for the investigators to identify individual persons, obtaining individual informed consent was neither required nor applicable.

### 2.5. Statistical Analyses

For each infection, the weekly burden was expressed as the number of cases per 100,000 population, both for the overall population and separately for the predefined age groups (0–4, 5–19, 20–59, and ≥60 years). Mean weekly burdens were calculated separately for the 2024/25 and 2025/26 epidemic seasons. Since data did not meet the assumption of Gaussian distribution (checked with the Shapiro–Wilk test), a non-parametric method was employed. Between-season differences in weekly burden were assessed using the Wilcoxon signed-rank test, with observations paired by corresponding epidemiological week, for the overall population and separately within each age group. All statistical tests were two-sided, and *p* < 0.05 was considered statistically significant. To evaluate between-season similarity in the temporal patterns of individual respiratory infections, independent of differences in their absolute magnitude, weekly burden values were additionally normalized to the season-specific maximum, with the peak set to 1. For each infection, the correspondence between the 2024/25 and 2025/26 trajectories was quantified using Spearman’s rank correlation coefficient (Rs), calculated across the corresponding epidemic weeks. Cross-correlation analysis was performed over lags of −8 to +8 weeks to identify potential temporal displacement between seasons; positive lags indicated that the 2025/26 trajectory occurred later relative to 2024/25. Temporal characteristics of the epidemic waves were further assessed by comparing peak timing, the direction of consecutive week-to-week changes, and the full width at half maximum (FWHM), defined as the number of consecutive weeks surrounding the peak during which the burden remained at ≥50% of the season-specific maximum. The proportion of consecutive weekly intervals showing concordant directions of change between seasons was calculated as an additional measure of similarity in epidemic dynamics.

Age-specific temporal patterns were evaluated separately for each infection and epidemic season. To assess whether epidemic waves followed similar temporal trajectories across age groups independently of differences in absolute burden, age-specific weekly series were normalized to their respective within-season maxima. Pairwise correspondence between age-specific trajectories was assessed using Spearman’s rank correlation coefficients calculated across corresponding epidemic weeks. Cross-correlation analysis over lags of −8 to +8 weeks was additionally used to identify potential temporal leads or delays between age groups. Particular attention was given to comparisons of the two pediatric groups (0–4 and 5–19 years) with adults aged ≥60 years to explore whether epidemic activity consistently preceded or followed that observed in the oldest age group. Peak epidemiological weeks were also compared across age groups as a complementary measure of temporal sequence. Lead–lag relationships were interpreted as temporal precedence only and not as evidence of the direction of transmission between age groups. Because weekly observations constituted serially ordered time-series data and only two epidemic seasons were available, correlation coefficients, cross-correlation coefficients, lag estimates, FWHM, direction-concordance measures, and age-specific lead–lag estimates were interpreted descriptively rather than subjected to conventional significance testing.

## 3. Results

### 3.1. Overall Population Burden

**Figure 1:**
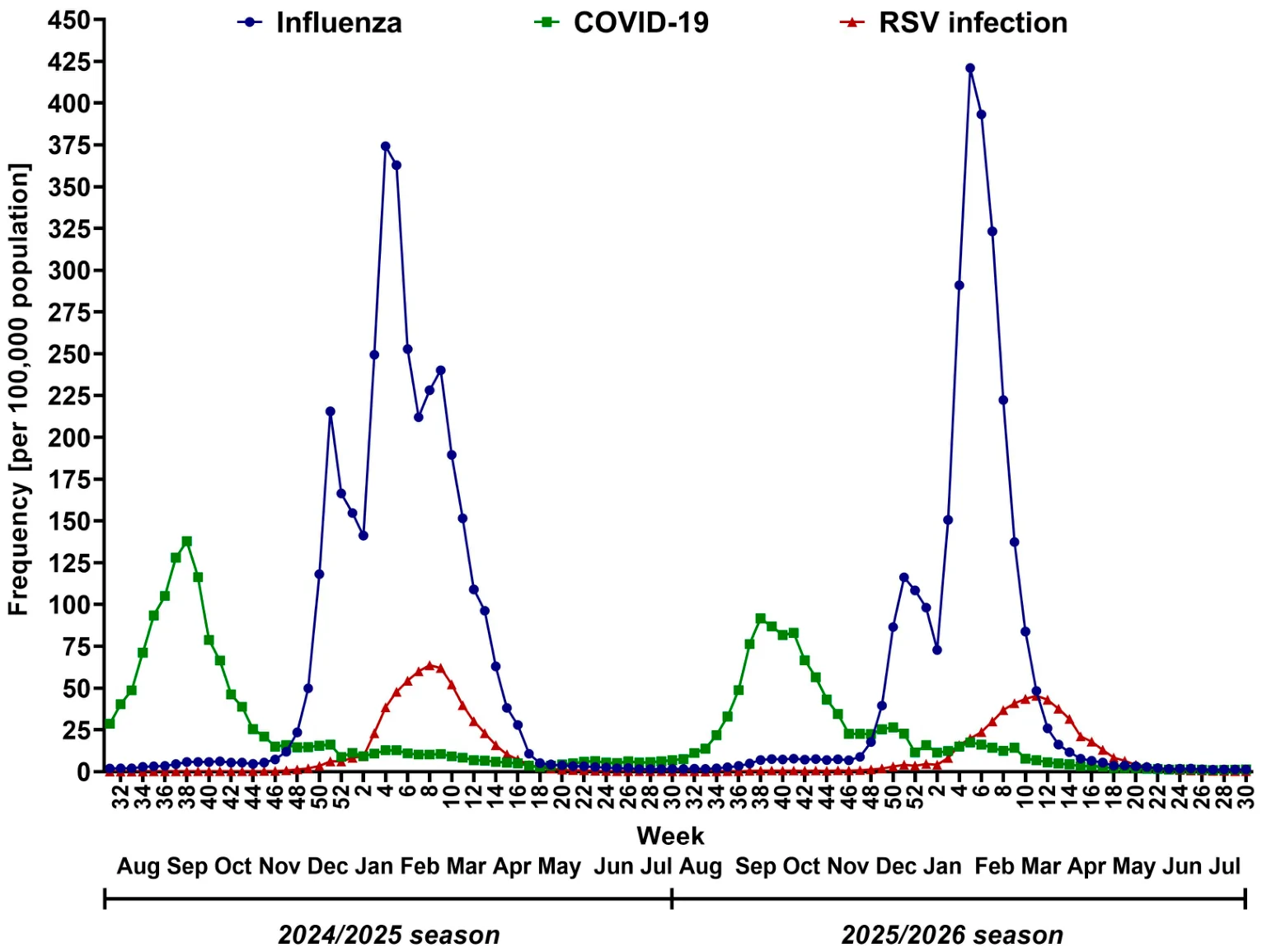
The overall burden of influenza (blue), COVID-19 (green), and RSV infections (red) in Poland in the 2024/25 and 2025/26 epidemic seasons.

The mean weekly burden of the analyzed infections over the entire epidemic season decreased in the following order: influenza (69.1 and 53.9 per 100,000 in 2024/25 and 2025/26, respectively) > COVID-19 (26.3 and 21.1 per 100,000) > RSV infections (11.2 and 9.4 per 100,000). The mean seasonal burden was numerically lower in 2025/26 for all three infections, by 22.0% for influenza, 19.5% for COVID-19, and 16.0% for RSV infections, although these between-season differences reached statistical significance only in the case of influenza (Wilcoxon signed-rank test, *p* = 0.0024). As shown in Figure 1, both seasons followed the same overall temporal sequence of infection waves, with COVID-19 preceding influenza and RSV infections. COVID-19 activity increased first, from August, and reached its maximum in week 38 (September) in both seasons. Influenza increased markedly from November/December and peaked in week 4 (January) of 2024/25 and week 5 (late January/early February) of 2025/26, whereas RSV infections peaked subsequently, in week 8 (February) and week 11 (March), respectively. Despite the lower mean seasonal burden in 2025/26, the peak influenza burden was 12.5% higher than in 2024/25 (421.0 vs. 374.2 per 100,000), whereas the peak burdens of COVID-19 and RSV infections were 33.5% (91.7 vs. 137.9 per 100,000) and 28.7% lower (45.4 vs. 63.7 per 100,000), respectively.

Beyond these differences in magnitude, the temporal trajectories showed substantial between-season similarity. Same-week Spearman correlations were highest for influenza (Rs = 0.976), followed by RSV infections (Rs = 0.956) and COVID-19 (Rs = 0.883). Influenza demonstrated particularly close temporal correspondence, with 84.3% of consecutive weekly changes occurring in the same direction and only a one-week shift in peak timing. However, its 2025/26 wave was more concentrated, with an FWHM of 5 weeks compared with 8 weeks in 2024/25, indicating a higher but narrower epidemic peak. COVID-19 peaked in the same epidemiological week in both seasons and showed similar wave widths (FWHM: 7 vs. 8 weeks), although its overall trajectory differed more than that of influenza, particularly in the configuration of the post-peak decline. RSV infections showed a highly similar temporal pattern but a clear delay in 2025/26: its peak occurred three weeks later, while the widths of the principal waves remained comparable (FWHM: 8 vs. 9 weeks). Cross-correlation analysis further supported this temporal displacement, with maximum correspondence obtained at a 3-week lag for RSV infections (r = 0.984). Overall, these findings indicate that the temporal architecture of the respiratory infection season was largely preserved across seasons, while individual infections differed in epidemic magnitude and, particularly for RSV infections, in peak timing.

### 3.2. Age-Stratified Burden

**Figure 2:**
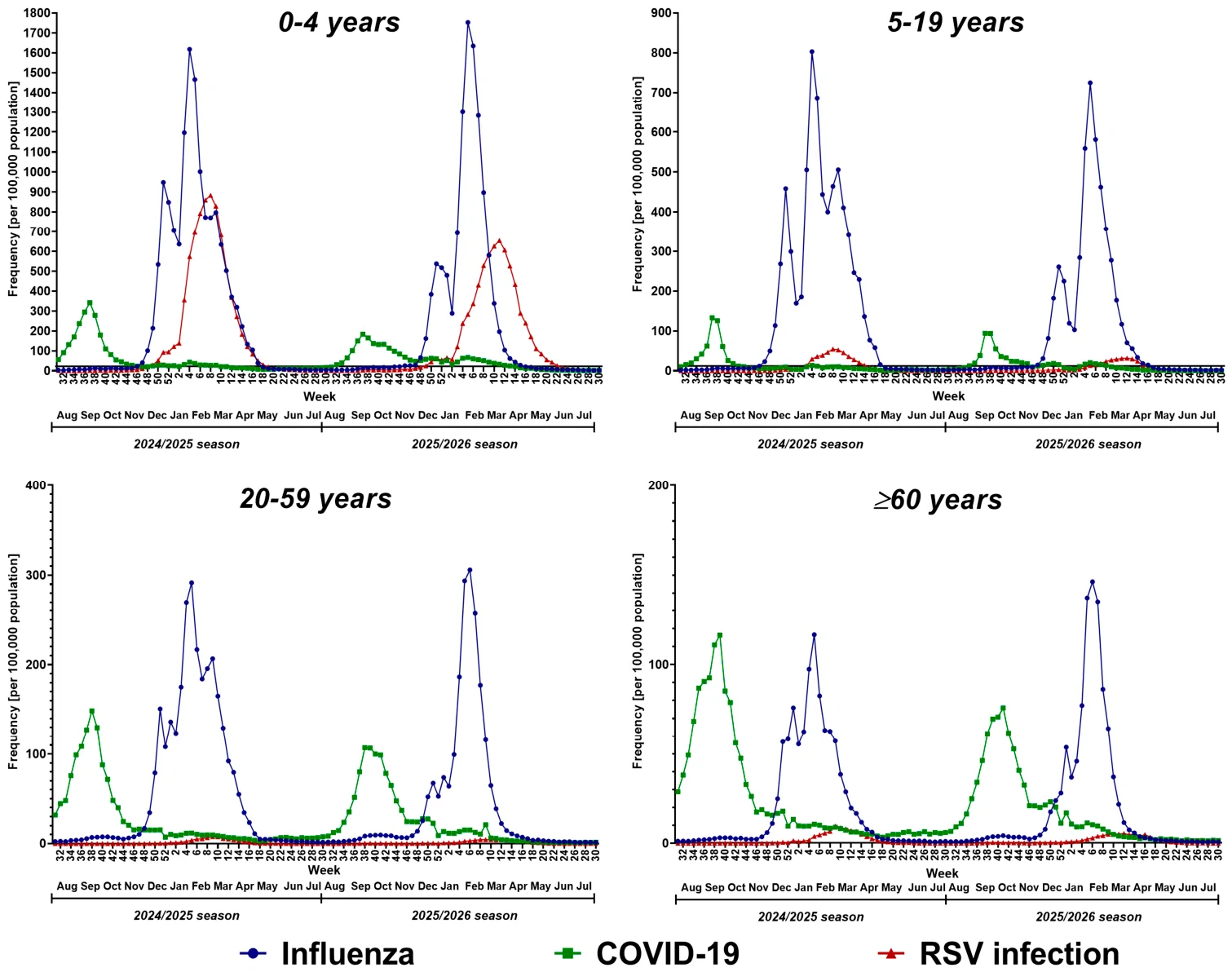
The age-stratified burden of RSV, influenza, and COVID-19 in Poland in the 2024/25 and 2025/26 epidemic seasons. Note the differences in the Y-axis range for particular age groups.

As shown in Figure 2, marked age-related differences were observed in the burden of all three respiratory infections.

Influenza burden decreased progressively with age, with the highest mean weekly rates observed among children aged 0–4 years (273.7 and 225.2 per 100,000 in 2024/25 and 2025/26, respectively), followed by those aged 5–19 years (134.8 and 93.9), adults aged 20–59 years (56.0 and 40.2), and adults aged ≥60 years (19.7 and 19.6). An even stronger age gradient was apparent for RSV infections, with the mean weekly burden among children aged 0–4 years (153.2 and 131.6 per 100,000) approximately 91-fold higher than among adults aged ≥60 years (1.68 and 1.44 per 100,000) in both seasons. In contrast, the age distribution of COVID-19 was less monotonic: the burden was highest in children aged 0–4 years (54.7 and 50.0 per 100,000), lowest in those aged 5–19 years (14.9 and 14.1), and intermediate in adults aged 20–59 years (27.3 and 23.0) and ≥60 years (25.6 and 17.2). For RSV, no significant between-season differences in weekly burden were observed in any age group (Wilcoxon signed-rank test, *p* > 0.05 for all comparisons). In contrast, influenza burden was significantly lower in 2025/26 than in 2024/25 across all age groups: 0–4 years (by 17.7%, *p* = 0.03), 5–19 years (by 30.3%, *p* = 0.004), 20–59 years (by 28.2%, *p* = 0.0009), and ≥60 years (by 1.0%, *p* = 0.04). For COVID-19, a significant between-season difference was observed only among adults aged ≥60 years (by 32.8%, *p* = 0.004), with a lower weekly burden in 2025/26, whereas no significant differences were observed in the remaining age groups.

**Figure 3:**
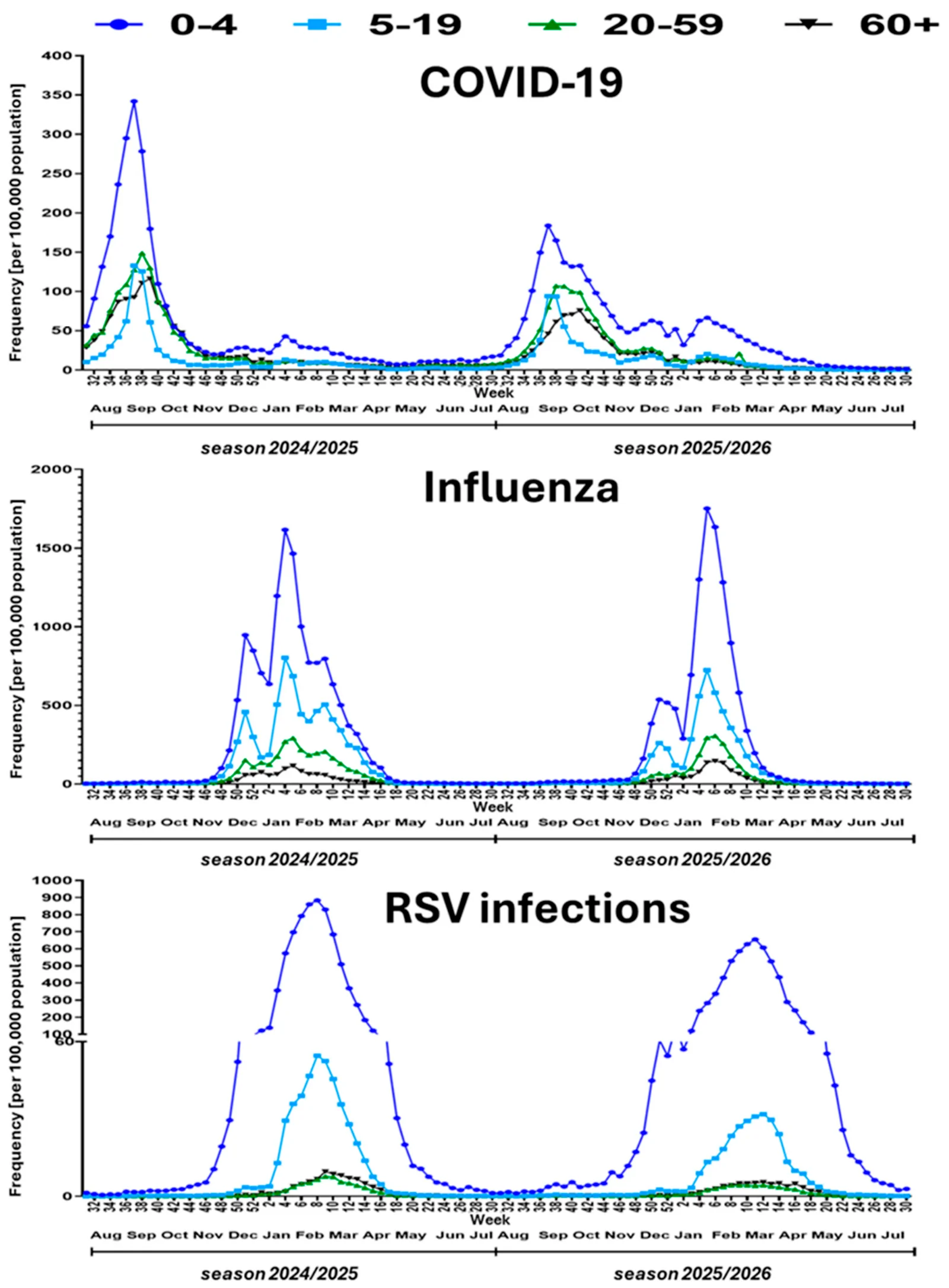
Age-specific temporal dynamics of COVID-19, influenza, and RSV infections during the 2024/25 and 2025/26 epidemic seasons, showing an apparent temporal lead in the 0–4-year age group. Note the differences in the Y-axis range for particular infections.

Despite substantial differences in absolute burden, the temporal patterns within each infection were highly concordant across age groups. For influenza, pairwise same-week correlations between age-specific trajectories were particularly high (Rs = 0.972–0.987 in 2024/25 and Rs = 0.963–0.992 in 2025/26), indicating an almost synchronous epidemic wave across the population. Children aged 0–4 and 5–19 years reached their influenza peaks in week 4 of 2024/25 and week 5 of 2025/26, approximately one week before adults aged 20–59 and ≥60 years; however, cross-correlation analysis generally identified no temporal displacement, or at most a 1-week lead in infection peak relative to the oldest age group. Notably, the ascending phase of the epidemic, particularly in children aged 0–4 years, became apparent earlier than in the adult groups, indicating a more pronounced pediatric lead during the initial increase than suggested by peak timing alone (Figure 3).

A more apparent age-related temporal sequence was observed for COVID-19, despite similarly high concordance between age-specific trajectories (pairwise same-week correlations: Rs = 0.875–0.987 in 2024/25 and Rs = 0.939–0.986 in 2025/26). In both seasons, the epidemic peak occurred first in the 0–4 and 5–19-year groups (week 37), followed by adults aged 20–59 years (week 38). Among adults aged ≥60 years, the peak occurred in week 39 in 2024/25 and week 41 in 2025/26. Cross-correlation analysis similarly indicated that the overall COVID-19 trajectory among children preceded that among adults aged ≥60 years by approximately 1 week in 2024/25 and 2 weeks in 2025/26. The rise in infection frequency was also apparent earlier in the 0–4-year group than in older adults, suggesting that age-related temporal separation was already present during the ascending phase of the epidemic and was not limited to differences in peak timing (Figure 3).

RSV also showed concordant age-specific temporal trajectories, although pairwise same-week correlations were lower than for influenza and COVID-19 (Rs = 0.826–0.944 in 2024/25 and Rs = 0.875–0.968 in 2025/26). In 2024/25, the peak among children aged 0–4 and 5–19 years occurred in week 8, compared with week 9 among adults aged ≥60 years, while cross-correlation indicated an approximately 2-week lead of the pediatric trajectories. In 2025/26, RSV peaked in week 11 among children aged 0–4 years and in week 12 among those aged 5–19 years and adults aged ≥60 years, corresponding to an approximately 1-week pediatric lead in cross-correlation analysis. Overall, the age-specific epidemic trajectories were strongly concordant for all three infections, but COVID-19 and, to a lesser extent, RSV consistently demonstrated earlier epidemic activity in younger age groups than in older adults, whereas influenza activity was largely synchronous across age groups. However, the temporal separation was considerably more apparent during the ascending phase: RSV activity in children aged 0–4 years began to increase well before a comparable rise was observed in the adult groups, particularly among those aged ≥60 years (Figure 3). Thus, the relatively small differences in peak timing underestimate the earlier emergence of RSV activity in young children.

## 4. Discussion

Our analysis of two consecutive post-pandemic respiratory infection seasons demonstrates a remarkably consistent temporal organization of influenza, COVID-19, and RSV circulation in Poland. In both 2024/25 and 2025/26, SARS-CoV-2 activity increased first, followed by influenza and subsequently RSV infections. Although the magnitude of individual waves differed between seasons, their overall temporal architecture was largely preserved. An important contribution of the present study is the joint characterization of these three major respiratory infections within a common nationwide population-level framework, made possible by the recently introduced national e-Health registry. This provides an updated reference for their relative timing, magnitude, and age-specific dynamics in the post-pandemic period and a benchmark against which patterns observed in subsequent seasons can be compared.

Rather than representing completely discrete seasonal entities, the analyzed pathogens formed an extended continuum of respiratory viral activity spanning from late summer through spring. This temporal organization has important implications for surveillance systems, clinical preparedness, and the timing of preventive interventions, which must increasingly account for a prolonged and partially overlapping period of respiratory infection activity. However, it should be noted that SARS-CoV-2 seasonality is not fixed or fully established, reflecting the relatively recent emergence of the virus in humans and the continuing influence of changing population immunity, viral evolution, behavioral factors, and environmental conditions [34,35]. This is also consistent with the present findings, as between-season concordance of temporal trajectories was lower for COVID-19 than for influenza or RSV infections, suggesting greater interseasonal variability in the timing and shape of SARS-CoV-2 activity. Accordingly, the early-autumn pattern observed consistently in the two seasons analyzed here should not necessarily be assumed to recur identically in future years. Previous analyses focusing on COVID-19 hospitalizations in Poland also highlighted a high degree of unpredictability of SARS-CoV-2 epidemiological dynamics [27,36].

Our analysis shows that SARS-CoV-2 infections in Poland began to rise already in August, with peak incidence observed around mid-September. Although this timing coincides with the late-summer period of increased travel and population mobility, the present data do not permit the determination of the factors responsible for this rise. This early wave consistently preceded subsequent influenza and RSV activity, suggesting a reproducible temporal ordering of major respiratory pathogens within the two seasons studied. Influenza activity then emerged as a second major wave, partially overlapping with declining or fluctuating COVID-19 transmission, whereas RSV activity increased later, extending into late winter or early spring. The comparison between seasons further showed that similarity in temporal organization did not imply similarity in epidemic magnitude. Mean weekly burdens were numerically lower in 2025/26 for all three infections; however, only for influenza did this difference reach statistical significance. At the same time, influenza produced a 12.5% higher peak in 2025/26 despite its lower mean seasonal burden, and its principal epidemic wave was narrower than in 2024/25. In contrast, the peak burdens of COVID-19 and RSV infections were substantially lower in 2025/26. RSV also showed a notable temporal displacement, with its maximum occurring approximately three weeks later than in the preceding season, while cross-correlation analysis indicated that the overall shape of the RSV infection trajectory remained highly similar. These observations illustrate that mean seasonal burden alone may obscure clinically relevant differences in the concentration, timing, and intensity of epidemic activity.

This temporal pattern has direct implications for vaccination strategy and public health preparedness. In particular, the consistently early rise in SARS-CoV-2 activity indicates that updated vaccines should be available sufficiently early to allow immunization before substantial autumn transmission begins. In recent epidemic seasons, updated COVID-19 vaccines were recommended for authorization by the European Medicines Agency as early as late July [37]. However, in several settings, including Poland, logistical and administrative delays meant that vaccination programs were initiated only after the seasonal COVID-19 peak had already occurred, e.g., in late October or early November [38]. In both seasons analyzed in the present study, SARS-CoV-2 activity began increasing in August and peaked around mid-September, illustrating the potential mismatch between epidemic timing and vaccine deployment. However, SARS-CoV-2 is a relatively recent human pathogen, and its seasonal dynamics may not yet be fully established or as predictable as those of influenza or RSV, which complicates the identification of a consistently optimal vaccination window. Moreover, the present analysis covered only two epidemic seasons. Therefore, our findings do not support defining a specific optimal date for vaccination but rather argue for accelerating regulatory, procurement, distribution, and organizational procedures so that updated vaccines can be made available sufficiently early to permit flexible deployment in response to emerging epidemiological trends. Such preparedness may increase the likelihood that vaccination precedes, rather than follows, a substantial rise in infections. Delayed deployment after the epidemic curve has already entered its rapid growth phase may substantially reduce the potential population-level benefit of seasonal vaccination. Moreover, it may further contribute to reluctance toward COVID-19 vaccination, whose uptake is already low in Poland, likely reflecting, among other factors, limited public trust in these vaccines, including among medical professionals [39].

Age-stratified analyses revealed pronounced differences in the absolute burden of infection. Influenza showed a clear inverse age gradient, with the highest rates among children aged 0–4 years and progressively lower rates in older groups. This gradient was even more striking for RSV infections, with the mean weekly burden among children aged 0–4 years approximately 91-fold higher than that among adults aged ≥60 years. COVID-19 displayed a different age distribution: rates were highest among children aged 0–4 years, lowest among those aged 5–19 years, and intermediate among adults. Thus, the age distribution of COVID-19 differed substantially from the monotonic patterns observed for influenza and RSV.

The age-specific temporal analyses also revealed important differences between pathogens. Influenza activity was highly synchronous across age groups, with very high same-week correlations and only minimal differences in peak timing. This suggests that influenza waves affected the different age strata largely in parallel rather than progressing sequentially through the population. In contrast, COVID-19 showed a more consistent age-related temporal sequence. In both seasons, the epidemic peak occurred first in the pediatric groups, followed by adults aged 20–59 years and subsequently by those aged ≥60 years. Cross-correlation analysis similarly indicated that COVID-19 activity among children preceded that in the oldest age group by approximately one week in 2024/25 and two weeks in 2025/26.

RSV demonstrated a similar, although more modest, pediatric lead. Its epidemic trajectory was highly concordant across age groups, but activity among children generally preceded that among adults aged ≥60 years by approximately one to two weeks. This observation has potentially important surveillance implications. Given the very high burden and greater diagnostic detectability of RSV in young children, increasing pediatric RSV activity may provide an early epidemiological signal of a subsequent increase among older adults. Such a sentinel role could be particularly useful because RSV remains substantially underdiagnosed in adults. This underdiagnosis is influenced, among other factors, by limitations of commonly used diagnostic approaches. In particular, rapid antigen tests have lower sensitivity in adults than in children, partly because of lower viral loads, and consequently, a negative antigen test does not exclude RSV infection in an older patient with a compatible clinical presentation [40].

However, the observed temporal precedence of pediatric COVID-19 and RSV activity should not be interpreted as direct evidence of transmission from children to older adults. The present study used aggregated population-level data and did not include household contacts, transmission pairs, viral sequencing, or individual-level exposure information. Therefore, our findings are compatible with, but do not prove, a role for children in initiating or amplifying transmission in older age groups. Shared environmental, behavioral, educational, or seasonal drivers could also contribute to the observed lag [41,42,43,44]. Likewise, transmission within households and the wider community is likely to occur in multiple directions rather than through a simple unidirectional pathway [45]. RSV nevertheless occupies a distinctive epidemiological position because its principal infection wave occurred after those of COVID-19 and influenza in both seasons. Its later timing means that substantial RSV activity may develop when healthcare services have already experienced several months of increased respiratory infection burden. A similar point was recently made in an analysis of the hospital burden caused by SARS-CoV-2, influenza viruses, and RSV in Poland [36]. This reinforces the need to maintain diagnostic awareness and preparedness beyond the influenza peak rather than assuming that respiratory virus activity declines uniformly after mid-winter.

It should be noted that RSV vaccination is currently available for adults and is mostly utilized in the elderly population [14,46]. The present findings reinforce the importance of protecting older adults, as recorded incidence substantially underestimates the clinical importance of RSV in this population: infection rates are lower than in young children, but the risk of severe lower respiratory tract disease rises markedly with age and comorbidity [47]. Vaccination of older adults should therefore primarily be viewed as direct protection against severe RSV disease rather than as an established strategy for preventing transmission to children. Such an effect, though plausible [48,49], would need to be tested in community settings. Protection of infants and young children relies on age-appropriate preventive approaches, including maternal vaccination [50] and long-acting monoclonal antibody prophylaxis [51]. Nevertheless, strengthening vaccination coverage and awareness among older adults should therefore remain a public health priority [52].

The age-specific lead–lag findings may also have practical value for integrated respiratory virus surveillance. The particularly early rise observed in children aged 0–4 years suggests that changes in this age group may provide an additional early signal of an emerging epidemic wave, potentially preceding differences apparent from peak timing alone. Pediatric surveillance could potentially provide an early warning signal for subsequent COVID-19 and RSV activity among older adults, whereas the near-synchronous influenza curves suggest that surveillance signals for influenza should be interpreted as reflecting population-wide activity rather than a clear sequential progression between age groups. Combining age-stratified surveillance across multiple respiratory pathogens may therefore improve anticipation of changes in healthcare demand and assist in the timing of preventive communication and vaccination programs.

Several limitations of this study should be acknowledged. First, while the e-Health Center database provides comprehensive national coverage, case ascertainment relies on routinely reported medical events, sick leave records, and laboratory testing, which may not capture all infections. In particular, rapid antigen tests, which are increasingly used in outpatient settings, exhibit high specificity for detecting RSV but variable sensitivity depending on patient age. Sensitivity is generally lower in adults than in children, likely due to differences in viral load dynamics, which may lead to underestimation of RSV infections, especially among elderly individuals [40]. Testing and healthcare-seeking behavior, as well as access to diagnostic testing, may also vary by age, pathogen, and epidemic season, potentially affecting comparisons of recorded incidence. Additionally, despite efforts to define unique cases by excluding events occurring within 60 days of a prior episode, some infections may still be misclassified. Second, only two epidemic seasons were available for comparison. Consequently, the consistency of the observed sequence and age-specific temporal relationships should be confirmed across additional seasons before they are regarded as stable epidemiological features. Third, weekly observations constitute serially ordered time-series data and may exhibit serial dependence due to the temporal continuity of epidemic waves. Although weekly values represented non-cumulative incidence estimates, such dependence may also affect the interpretation of the Wilcoxon signed-rank test if it is present in the paired between-season differences, potentially reducing the effective amount of independent information and resulting in overly optimistic nominal *p*-values. The Wilcoxon test results should therefore be interpreted with appropriate caution. For the same reason, correlation, cross-correlation, peak timing, and lead–lag estimates were interpreted descriptively rather than as conventional independent-observation tests. Most importantly, temporal precedence between age groups cannot establish the direction or mechanism of transmission. Finally, although the national database provides population-level surveillance estimates, the analytical dataset available for the present study did not contain the individual-level information required to characterize cases according to detailed clinical presentation (e.g., upper versus lower respiratory tract infection) or the simultaneous detection of more than one of the three analyzed viruses. Consequently, sex-specific patterns, clinical manifestations, and viral co-infections could not be evaluated. Similarly, because the study was based on population-level surveillance rates rather than a recruited cohort, the distribution of individual participants across demographic or clinical categories beyond the predefined age groups could not be reported. The study also did not include data on vaccination status, circulating viral subtypes/lineages, healthcare utilization, hospitalizations, or detailed information on disease severity, comorbidities, or deaths, as these variables were not available in the dataset used for the present analysis. These limitations restrict the interpretation of the findings to population-level temporal and age-specific epidemiological patterns and preclude conclusions regarding individual-level clinical characteristics or co-infection patterns. We advocate expanding it to include these variables, as this would allow for a more precise assessment of disease burden and calculation of vaccine effectiveness.

## 5. Conclusions

Our analysis of respiratory infection dynamics in Poland during the 2024/25 and 2025/26 seasons revealed a consistent temporal sequence of COVID-19, followed by influenza and subsequently RSV infections, despite between-season differences in the magnitude and timing of individual epidemic waves. COVID-19 activity had already begun to increase in August and peaked around mid-September in both seasons, highlighting a potentially important mismatch between epidemic timing and the deployment of updated vaccines. As they may become available only shortly before, or even after, the onset of the seasonal increase in infections, regulatory, procurement, distribution, and organizational procedures should be optimized to enable vaccination to begin earlier, allowing sufficient time for protection to develop before the rise in transmission. Influenza showed the greatest temporal synchrony across age groups, whereas COVID-19 and RSV infections demonstrated modest but consistent earlier activity among children than among adults aged ≥60 years. However, across all three infections, the ascending phase appeared particularly early in children aged 0–4 years, suggesting that peak timing alone may underestimate the pediatric temporal lead. Although these lead–lag relationships cannot establish the direction of transmission, they suggest that pediatric surveillance may provide an early indication of subsequent increases in COVID-19 and RSV activity among older populations. The pronounced age gradients in influenza and particularly RSV burden further emphasize the need for age-targeted preventive strategies. Integrated, age-stratified surveillance of COVID-19, influenza, and RSV infections, combined with earlier, better-aligned vaccination programs and appropriate diagnostic preparedness, may improve the response to the prolonged, partially overlapping respiratory infection season.

## Data Availability

The raw data analyzed in this study are available in the e-Health Center at https://ezdrowie.gov.pl/, accessed on 15 August 2026.
